# DNA mismatch repair (MMR) genes expression in lung cancer and its correlation with different clinicopathologic parameters

**DOI:** 10.1038/s41598-024-83067-2

**Published:** 2025-01-06

**Authors:** Mayada Saad Farrag, Heba Wagih Abdelwahab, Amr Abdellateef, Nahla Anber, Mohamed Adel Ellayeh, Dalia Tawfeek Hussein, Ahmed Ramadan Eldesoky, Heba Sheta

**Affiliations:** 1 Pathology Department, Port Said Faculty of Medicine, Port Said University, Port Said, Egypt; 2 Chest Medicine Department, Mansoura Faculty of Medicine, Mansoura, Egypt; 3 Cardiothoracic Surgery Department, Faculty of Medicine, Mansoura University, Mansoura, Egypt; 4 Emergency Hospital, Mansour University, Mansoura, Egypt; 5 Children’s Hospital, Faculty of Medicine, Mansoura University, Mansoura, Egypt; 6 Faculty of Medicine, Tobruk University, Tobruk, Libya; 7 Clinical Oncology and Nuclear Medicine Department, Mansoura Faculty of Medicine, Mansoura, Egypt; 8 Pathology Department, Faculty of Medicine, Mansoura University, Mansoura, Egypt

**Keywords:** Lung cancer, MMR, MLH1, MSH2, MSH6, PMS2, Cancer, Medical research, Oncology, Pathogenesis

## Abstract

Lung cancer (LC) is a crucial rapidly developing disease. In Egypt, it is one of the five most frequent cancers. Little is known about the impact of deleted mismatch repair genes and its correlation to clinicopathological characteristics. This study evaluates immunohistochemical expression of the mismatch repair genes (PMS2), (MSH2), (MLH1) & (MSH6) & its correlation with clinicopathologic parameters & prognosis of LC. Age was higher with lost MLH1 & PMS2 but HTN was higher with lost four markers. Smoking was associated with expression of MLH1 & PMS2. A progressive course was associated with lost MSH2 & MSH6. Suprarenal metastasis was associated with lost all markers but bone metastasis was associated with lost MSH2 & MSH6. All the markers were significantly correlated with each other, with perfect correlations between MSH6 & MSH2 and between MLH & PMS2. Median overall survival among cases with lost markers was significantly lower than patients with preserved markers. We recommend evaluation of the four proteins as a biomarker that could guide LC therapy. In-depth biological research is imperative to elucidate the precise roles and mechanisms of these markers. This will advance management strategies and even guide immune checkpoint inhibitor therapy for LC.

## Introduction

Lung cancer (LC) is a crucial rapidly developing illness that poses a global health challenge. Globally, it is classified as the 2nd most frequent malignant tumor, and regarding GLOBOCAN 2020 database estimates, it is the main etiology of cancer-related deaths, accounting for 18% of all deaths by cancer^1^. In Egypt, LC is one of the five most frequent cancers, with an estimated 5-year prevalence of 6.95/105 and an incidence of 4.9% in 2020^2^.

In Western societies, approximately half of all LC patient have been diagnosed with metastatic illness^3^. Nonetheless, there is a few information available regarding the characteristics of Arab or Egyptian cases with lung cancer^4^. Despite significant improvements in diagnostic approaches and efficient modern management strategies, the lung cancer 5-year survival rate is only ten to twenty% due to poor prognosis and late diagnosis^5^. Over 75% of LC are diagnosed late in more advanced stages with multiple systemic metastases, especially in developing nations^6^. Poor LC prognosis is attributed mainly to the lack of adequate screening methods^7^.

Small cell lung cancer (SCLC) & Non-small cell lung cancer (NSCLC) are the main pathologic forms of lung cancer. NSCLC accounts roughly for 85% of all LC patients^8^. There are several unique characteristics of LC^9^. Pathological investigation of LC tissues is fundamental to diagnosis, management decisions & prognosis. Improved understanding of the heterogeneous tumor microenvironment may provide new avenues for LC management^10^.

The four principal DNA repair processes are DNA mismatch repair (MMR), double-strand break repair (DSBR), base excision repair (BER)& nucleotide excision repair (NER). DNA mismatch repair is an essential repair mechanism that initiates the DNA damage response^11^. It is essential for the maintenance of genome homeostasis to be able to correct errors during DNA replication^12^. Certain protein complexes recognize and metabolize mismatches in DNA. Eventually, the mismatches are filled in^13^.

Proficient mismatch repair (pMMR) refers to the state of preservation or maintenance of the MMR function, while deficient mismatch repair (dMMR) represents the reduction of the DNA mis match repair function. The number of repeats of one-base to several-base repeat sequences is altered by the reduced repair function. Short tandem repeats are also known as microsatellites. Microsatellite instability (MSI) is the term that is utilized to describe this phenomenon. The tumorigenesis process is believed to be significantly motivated by the accumulation of these genomic alterations^12^. Little is known about the impact of dMMR and its correlation with clinicopathological characteristics and overall survival (OS) in Egyptian or even in Arab LC patients.

To our knowledge, the current research is one of the most important and insightful LC studies. It was conducted retrospectively on Egyptian LC patients to enhance our current knowledge about LC. We evaluated the immunohistochemical expression of the MMR genes, postmeiotic segregation increased 2 (PMS2), mutl homolog 2 (MSH2), mutl homolog 1 (MLH1) & mutl homolog 6 (MSH6). It distinguishes between their expression in various histopathological forms of lung cancer. It also establishes a correlation between these genes’ expression and a variety of clinicopathologic parameters & also its association with prognosis of lung cancer. Thus, we can study the impact of MMR genes and proteins differential expression on LC patient prognosis.

## Materials and methods

The present study is retrospective in nature. It comprises 38 biopsy specimens that were obtained through bronchoscopy, CT-guided procedures or surgical maneuvers. In collaboration between the chest medicine department, the Oncology Center, the cardiothoracic surgery department& the Clinical Oncology & Nuclear Medicine department, the diagnosis of lung cancer cases was made through biopsies sent to pathology departments at Mansoura University, Mansoura, Egypt, throughout the duration from 2018 to 2022. This research has been permitted by the institutional research board of the Faculty of Medicine at Mansoura University (R.22.09.1847). All methods were performed in accordance with the relevant guidelines and regulations.

A random selection of cases was performed. Utilizing Excel software, the final sample was selected through simple random sampling. Using the same methodology, any patient selected with an absent paraffin block was substituted with another. We gathered the relevant clinicopathologic data. We also monitored the clinical outcomes of the cases in the form of progression-free survival (PFS)& overall survival (OS). Duration from diagnosis to mortality was considered the overall survival. Duration from management initiation to the first recurrence, metastasis, or mortality is referred to as progression-free survival. To evaluate the adequacy of tumor tissue for immunostaining, hematoxylin & eosin (H&E) slides of the pathologic samples have been prepared & examined.

### Interventional procedures

Central lesions were sampled by a bronchoscopic transbronchial lung biopsy (either by forceps or cryo-biopsy). Peripheral lesions were sampled by a tru-cut needle with CT guidance at the chest medicine department. Patients whose bronchoscopic or CT-guided biopsies came as “non-diagnostic” and patients who were candidates for definitive surgery underwent thoracoscopic, surgical biopsy or resection at the cardiothoracic surgery department. Those surgical procedures had been done under general anesthesia with double lumen endotracheal intubation via a uni-portal thoracoscopic approach or conventional thoracotomy. A frozen section was prepared intraoperatively to guarantee significant tissue biopsies.

### Immunohistochemical staining

Four-micrometer-thick slices have been cut from all specimens. Mouse monoclonal antibodies against mutl homolog 2 (MSH2) (ready to be utilized; clone fe11; dako/agilent), mutl homolog 1 (MLH1) (ready to utilize; clone g168-15; dako/agilent), post meiotic segregation increased 2 (PMS2) (ready to utilize; clone a16-4; Ventana/Roche) have been utilized to incubate the slides. As regards mutl homolog 6 (MSH6), a rabbit monoclonal antibody (ready to be utilized; clone sp93; Ventana/Roche) has been used following the manufacturer’s guidelines. Suitable positive and negative controls were prepared for all markers.

### Immunohistochemical assessment

The presence of nuclear staining in all tumor cells was recorded as positive expression. Tumor cells that exhibited complete loss of staining have been scored as negative for expression, provided that the normal cells surrounding the tumor exhibited nuclear staining. The criteria of staining heterogeneity were specified by Joost et al. (2014). Heterogeneous staining is characterized by tumors exhibiting intraglandular heterogeneity (featuring a mixture of strongly immunoreactive cells & negative cells) and/or zonal loss which involves confluent regions of staining deficiency over numerous neighboring glands^14,15^.

### Statistical analysis

SPSS software V.26 has been utilized to analyze the data. Frequencies and percentages have been used to represent categorical data. The assumption of a normal data distribution was tested using Shapiro-Wilk tests, which resulted in the presentation of continuous data as the mean slandered deviation or median (minimum-maximum). The Mann-Whitney U or median test, has been utilized to conduct statistical significance testing on continuous data. While categorical data were subjected to the chi-square test or Fisher’s exact test, as appropriate, The Kaplan-Meier test has been utilized to evaluate the overall survival and progression-free survival of patients correlates to the expression of MMR genes in their tumors. Log rank (Mantel-Cox) has been to conduct the comparison. The significance level has been established at 0.05.

## Results

### Cases clinicopathological characteristics

Regarding clinical characteristics of patients, Table 1 demonstrates that the study included 38 patients with a mean age of 62.5 (SD:1). Nearly 74% of them were males. Comorbidities were represented in 47.4% of studied patients: nearly 26% had hypertension (HTN), nearly 16% of them had diabetes mellitus (DM), and 15.8% had other lesser-represented comorbidities. Most studied patients presented with chest symptoms, mainly cough (71%), shortness of breath (58%), and chest pain (29%), in addition to 45% diagnosed at stage of pulmonary metastases. While 30% of the patients presented with nervous manifestations that were identified as brain metastases, 16% had renal manifestations diagnosed as suprarenal metastases. In addition, 42% were diagnosed as bone metastases. Nearly 43% of them were current smokers.

**Table 1:** characteristics of all patients.

Parameter		Frequency	Percent
Age	Mean (SD)	62.47 (10)	
Gender	Male	28	73.7
	Female	10	26.3
Diabetes	No	32	84.2
	Yes	6	15.8
HTN	No	28	73.7
	Yes	10	26.3
Comorbidities	No	20	52.6
	Yes	18	47.4
Smoking	No	14	37.8
	Ex	7	18.9
	Current	16	43.2
Pathological type	Adenocarcinoma	16	42.1
	Large cell NE carcinoma	1	2.6
	Squamous cell carcinoma	11	28.9
	SCLC	10	26.3
Grade	Moderate	12	31.6
	Poor	26	68.4
Side	Right	26	72.2
	Left	10	27.8
	Total		94.7
Site	Hilar	15	41.7
	Upper and lower lobes	3	8.3
	Upper	11	30.6
	Lower	6	16.7
	Chest wall mass	1	2.8
Course	Regressive	5	14.7
	Stationary	16	47.1
	Progressive	13	38.2
T stage	1	2	5.3
	2	9	23.7
	3	9	23.7
	4	18	47.4
N stage	0	8	21.1
	1	2	5.3
	2	15	39.5
	3	13	34.2
M stage	m0	6	15.8
	m1a	6	15.8
	m1b	4	10.5
	m1c	22	57.9
Stage group	IIA	1	2.6
	IIB	1	2.6
	IIIB	3	7.9
	IVA	8	21.1
	IVB	25	65.8
Lost MLH1		3	7.8%
Lost PMS2		3	7.8%
Lost MSH6		4	10.5%
Lost MSH2		4	10.5%

**Fig. 1:**
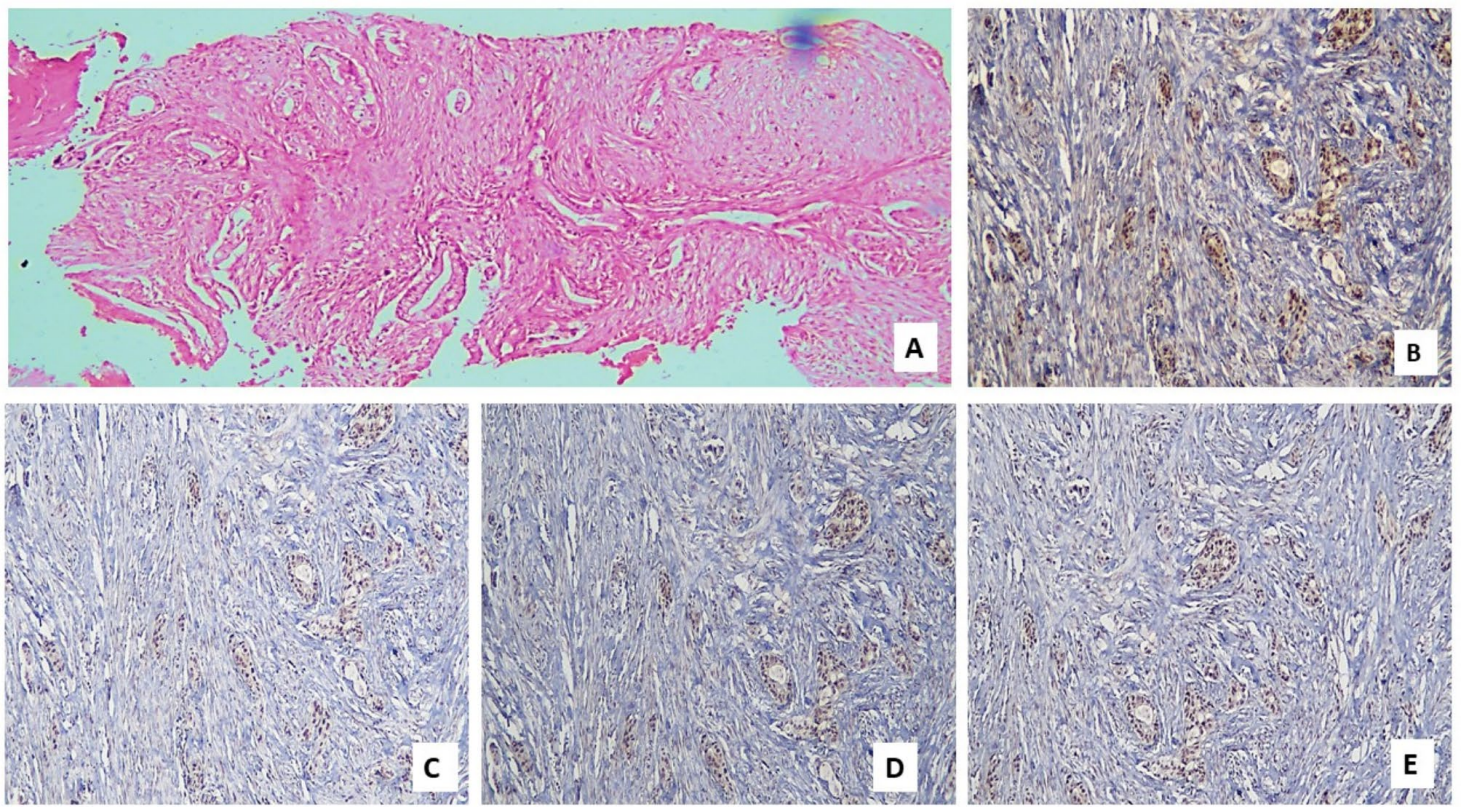
Photomicrograph of a case of adenocarcinoma with proficient (positive) MMR genes expression: (**A**) Malignant tumoral proliferation arranged in glandular architecture; lined by malignant epithelial cells and separated by desmoplastic stroma (Hx & E; X100). (**B**) Proficient nuclear IHC expression of tumor cells for MLH1 (X 200). (**C**) Proficient nuclear expression of tumor cells for PMS2 (X 200). (**D**) Proficient nuclear expression of tumor cells for MSH2 (X 100). (**E**) Proficient nuclear expression of tumor cells for MSH6 (Magnification X: 200).

**Fig. 2:**
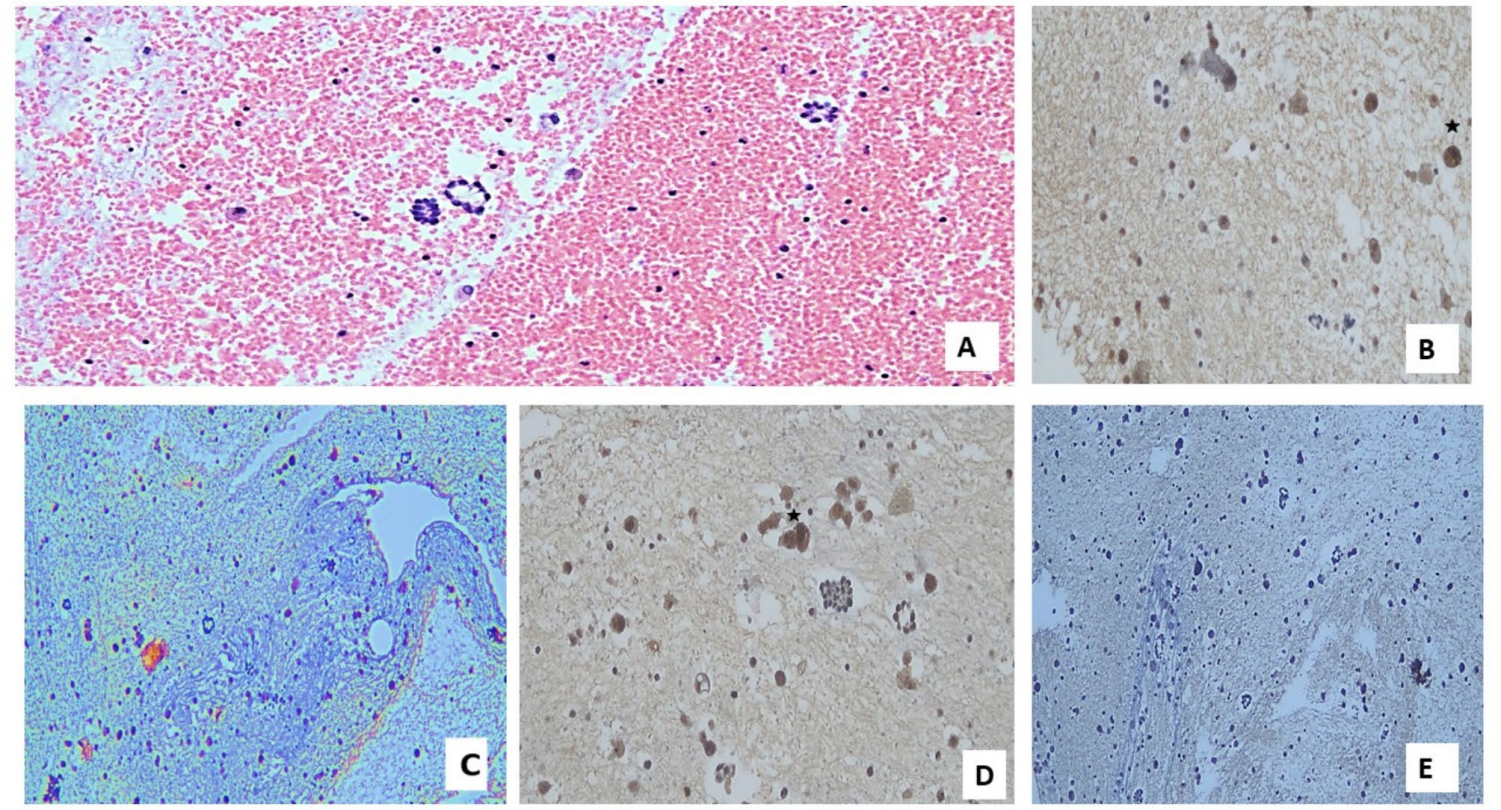
Photomicrograph of a cell block of pleural effusion in a case of adenocarcinoma with deficient (negative) MMR genes expression: (**A**) Groups of atypical cells that form papillary structures admixed with reactive mesothelial cells (Hx & E; X200). (**B**) Deficient IHC expression of tumor cells for MLH1 with positive internal control (reactive mesothelial cells) (star) (X200). (**C**) Deficient IHC expression of tumor cells for PMS2 (X100). (**D**) Deficient IHC expression of tumor cells for MSH2 with positive internal control in reactive mesothelial cells (star) (X200). (**E**) Deficient IHC expression of tumor cells for MSH6 (Magnification X: 100).

**Fig. 3:**
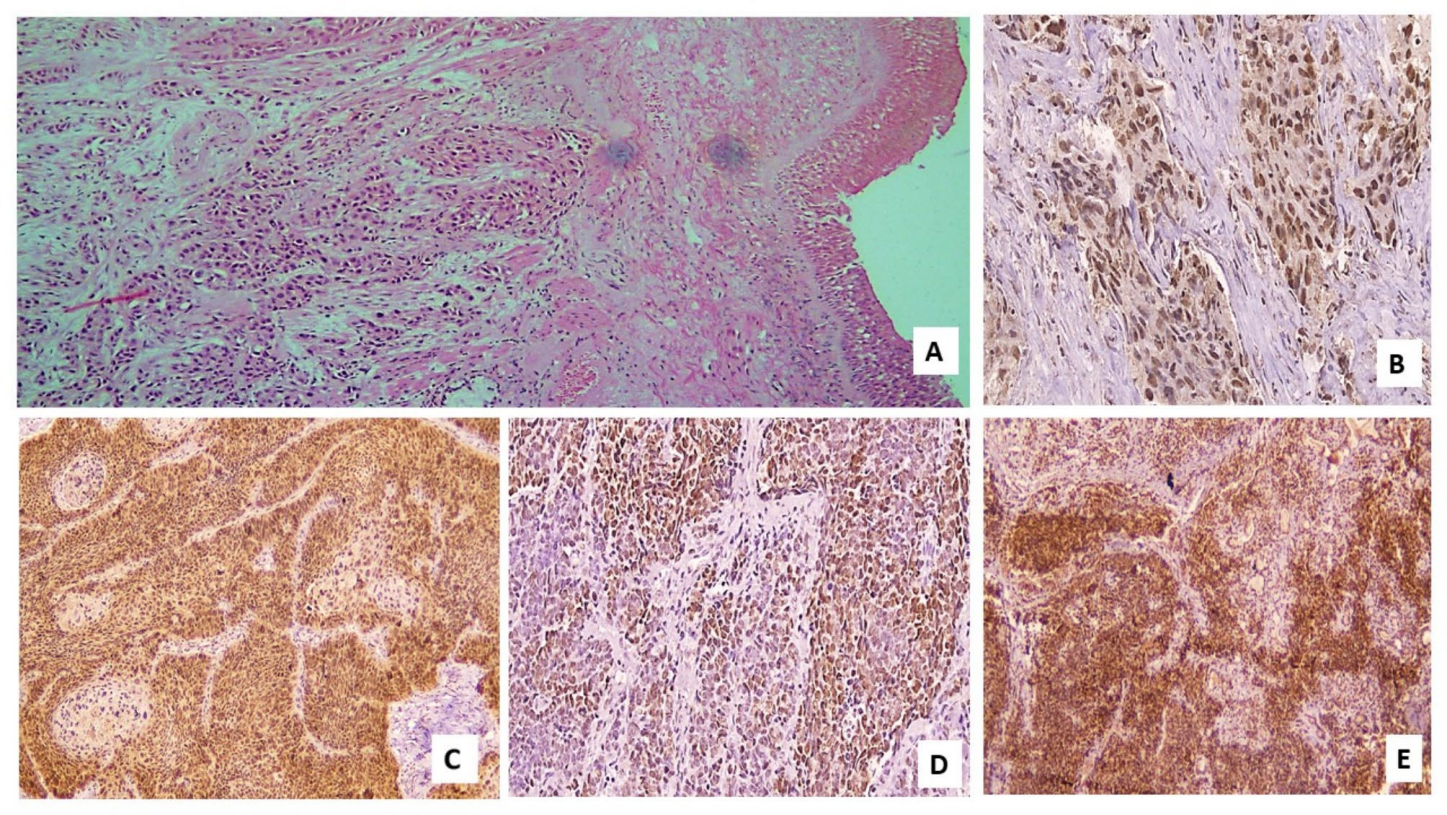
Photomicrograph of a case of SCC with proficient (positive) MMR genes expression: (**A**) Poorly differentiated squamous cell carcinoma with associated squamous metaplasia of the covering epithelium (Hx & E; X100). (**B**) Proficient IHC nuclear expression of tumor cells for MLH1 (X400). (**C**) Proficient nuclear expression of tumor cells for PMS2 (X200). (**D**) Proficient nuclear expression of tumor cells for MSH2 (X200). (**E**) Proficient nuclear expression of tumor cells for MSH6 (Magnification X:100).

**Fig. 4:**
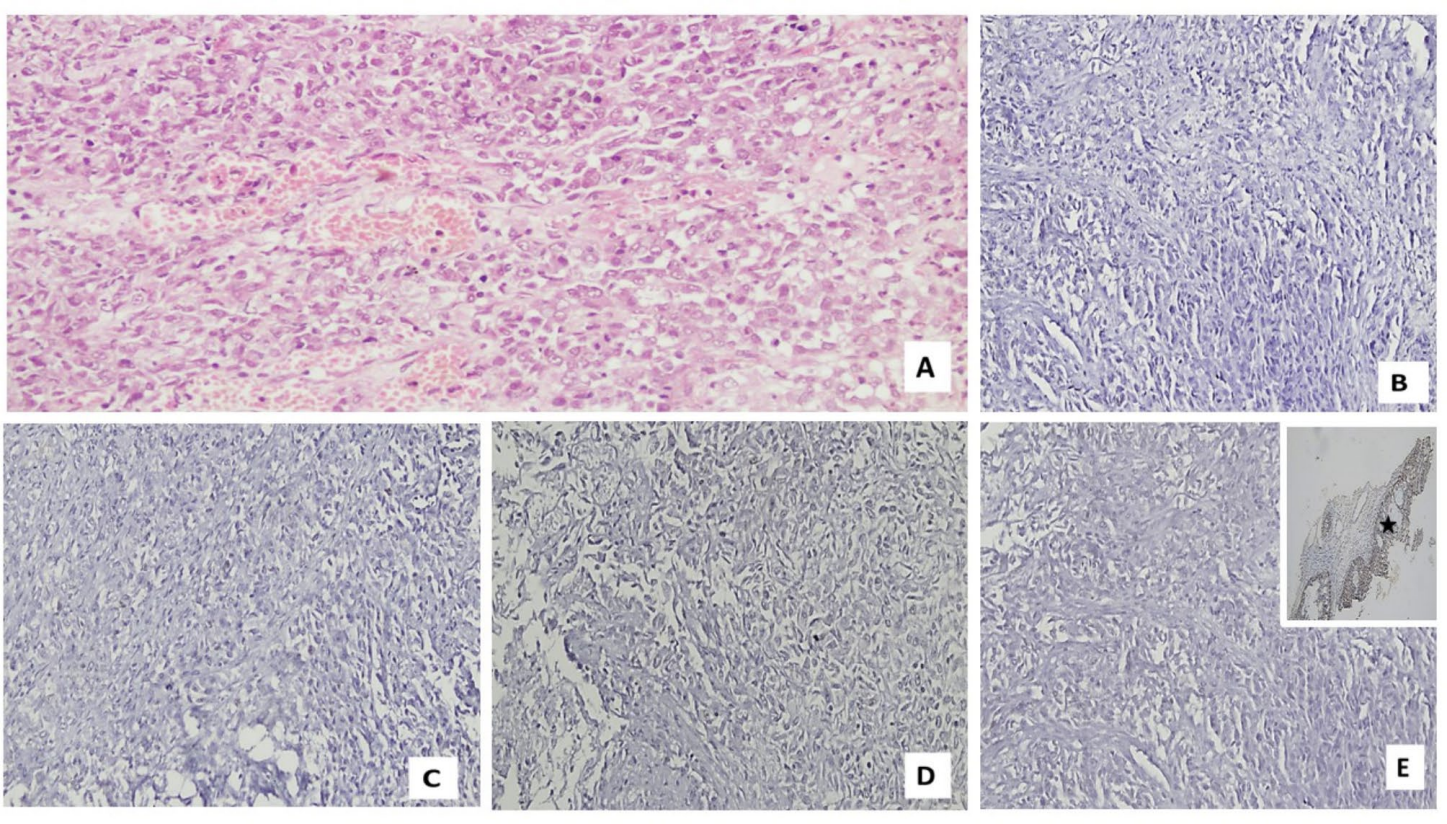
Photomicrograph of a case of SCC with deficient (negative) MMR genes expression: (**A**) Poorly differentiated squamous cell carcinoma with sheets of malignant squamous cells (Hx & E; X200). (**B**) Deficient nuclear expression of tumor cells for MLH1 (X200). (**C**) Deficient nuclear expression of tumor cells for PMS2 (X200). (**D**) Deficient nuclear expression of tumor cells for MSH2 (X200). (**E**) Deficient nuclear expression of tumor cells for MSH6 (Inset: positive expression of MLH1 in surface epithelium of the case as an internal control(star)) (Magnification X: 200).

**Fig. 5:**
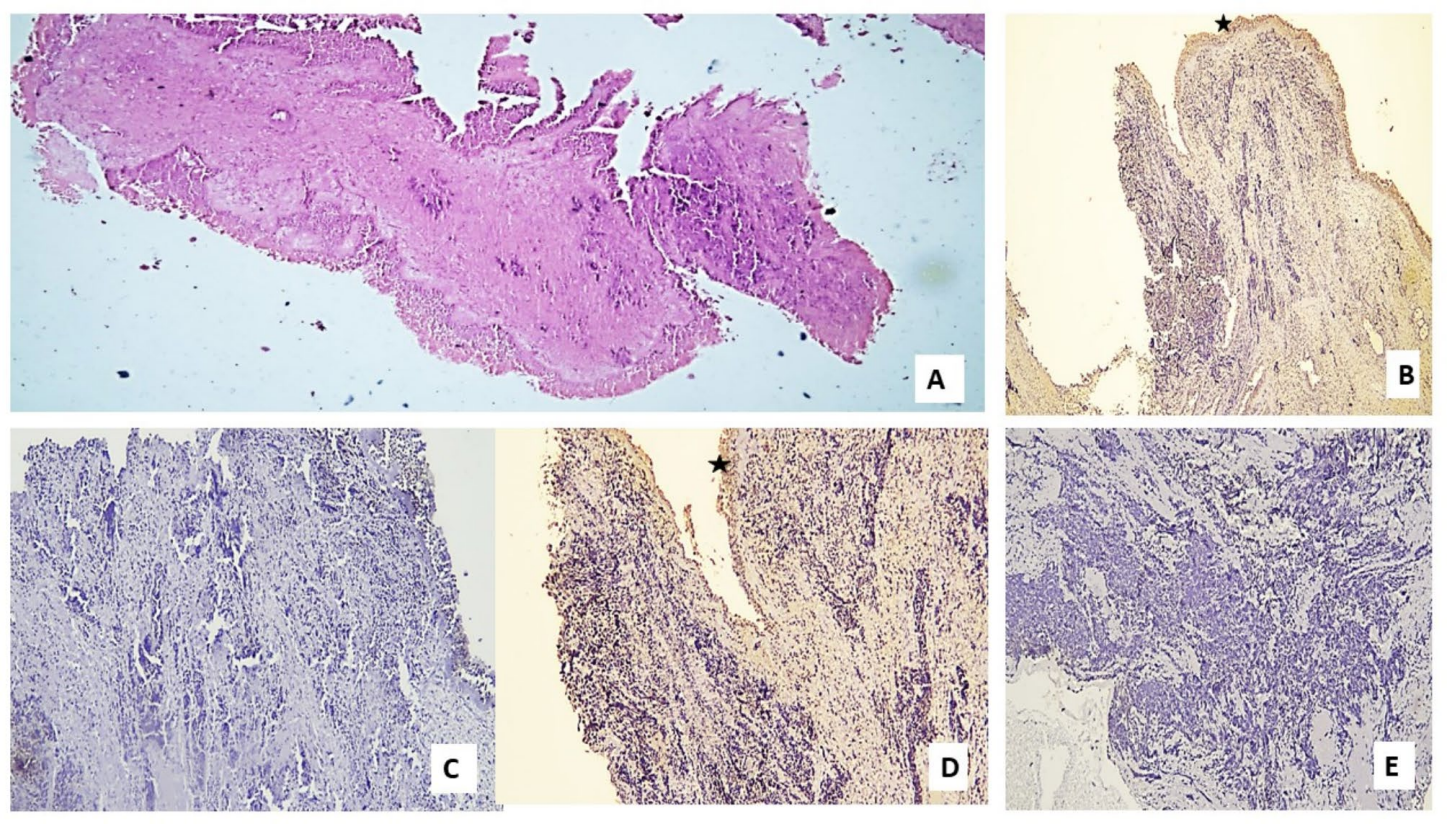
Photomicrograph of a case of small cell carcinoma (SCLC) with deficient (negative) MMR genes expression: (**A**) Infiltration by malignant tumoral proliferation formed of small blue round cells with crushing artifact (Hx & E; X40). (**B**) Deficient IHC expression of tumor cells for MLH1 (X100) with positive internal control in surface epithelium (star). (**C**) Deficient IHC expression of tumor cells for PMS2 (X200). (**D**) Deficient IHC expression of tumor cells for MSH2 with positive internal control in surface epithelium (star) (X200). (**E**) Deficient IHC expression of tumor cells for MSH6 (Magnification X: 200).

As regards pathological features, nearly 42% of cases had adenocarcinoma (Figs. 1A, 2A), nearly one-third had squamous cell carcinoma (28.9%) (Fig. 3A, 4A) and small cell lung cancer (SCLC) was found in 26.3% of cases (Fig. 5A). Regarding tumor grade, nearly 70% of patients were poorly differentiated (grade 3). The most common site was hilar (41.7%). Regarding tumor stage, most tumors were T2& T3 (23.7% for each of them). Most tumors were N2& N3 (38.5 & 34.2%, respectively). Stage M1c accounted for 57.9%of cases. The most common TNM stage was IV B (56.8%). As regards expression of MMR genes in the study cases, only 7.8% of patients had lost MLH1 and PMS2 markers, while 10.5% of them had lost MSH2 and MSH6 markers.

### Relationship between the expression of MMR genes and other clinicopathological characteristics

Table 2 demonstrates the correlation between MLH1 marker and characteristics of the cases. Age was significantly greater among the group with the lost marker (p-value equal 0.015). Also, HTN was significantly greater among the group with lost marker (p-value equal 0.014). Smoking was also associated with MLH1 marker (*p* = 0.045). Suprarenal metastasis was associated with lost MLH1 marker (*p* = 0.003). Apart from the previously mentioned parameters, none of the other characteristics of patients were associated with the marker (Figs. 1B, 2B, 3B, 4B and 5B).

**Table 2:** Association of MLH1 marker expression and the clinicopathological characteristics of patients.

			MLH1		p value
			Lost N = 3	Preserved N = 35	
Age	Mean (SD)		75.7 (5.5)	61.3(9.4)	t:2.6, **p:0.015**
Gender	Male	N%	2(7.1%)	26(92.9%)	1
	Female	N%	1(10.0%)	9(90.0%)	
Diabetes	No	N(%)	1(3.1%)	31(96.9%)	0.059
	Yes	N(%)	2(33.3%)	4(66.7%)	
HTN	No	N(%)	0(0.0%)	28(100.0%)	**0.014**
	Yes	N(%)	3(30.0%)	7(70.0%)	
Comorbidities	No	N(%)	0(0.0)	20(100.0)	0.097
	Yes	N(%)	3(16.7)	15(83.3)	
Smoking	No	N(%)	1(7.1)	13(92.9)	.**045**^**b**^
	Ex	N(%)	2(28.6)	5(71.4)	
	Current	N(%)	0(0.0)	16(100.0)	
Pathological type	Adenocarcinoma	N(%)	1(6.3)	15(93.8)	0.489
	Large cell NE carcinoma	N(%)	0(0.0)	1(100)	
	Squamous cell carcinoma	N(%)	2(18.2)	9(81.8)	
	SCLC	N(%)	0(0.0)	10(100)	
Grade	Moderate	N(%)	1(8.3)	11(91.7)	1
	Poor	N(%)	2(7.7)	24(92.3)	
Side	Right	N(%)	1(3.8)	25(96.2)	0.181
	Left	N(%)	2(20.0)	8(80.0)	
Site	Hilar	N(%)	1(6.7)	14(93.3)	0.832
	Upper and lower lobes	N(%)	0(0.0)	3(100)	
	Upper	N(%)	1(9.1)	10(90.9)	
	Lower	N(%)	1(16.7)	5(83.3)	
	Chest wall mass	N(%)	0(0.0)	1(100)	
Course	Stationary/regressive	N(%)	0(0.0)	21(0.0)	0.139
	Progressive	N(%)	2(15.4)	11(84.6)	
N stage	0/1	N(%)	0(0.0)	10(100)	0.552
	2/3	N(%)	3(10.7)	25(89.3)	
T stage	T1/2	N(%)	1(9.1)	10(90.9)	1
	T3/4	N(%)	2(7.4)	25(92.6)	
M stage	m0	N(%)	0(0.0)	6(100)	1
	M1A, B,C	N(%)	3(9.4)	29(90.6)	
Bone mets	No	N(%)	0(0.0)	22(100)	0.066
	Yes	N(%)	3(18.8)	13(81.2)	
Suprarenal mets	No	N(%)	0(0.0)	31(100)	**0.003**
	Yes	N(%)	3(50)	3(50)	
Stage group	II, III	N(%)	0(0.0)	5(100)	1
	IV	N(%)	3(9.1)	30(90.9)	

Bold indicates statistically significanct result where the significance level has been established at 0.05.

Table 3 shows the association of PMS2 marker & characteristics of cases. Age was significantly higher among the group with lost marker (p-value equal 0.025). Also, HTN was significantly greater among the group with lost marker (*p* = 0.014). Smoking was also associated with PMS2 marker (p-value equal 0.040). Suprarenal metastasis was associated with lost PMS2marker (p-value equal 0.003). Apart from the previously mentioned parameters, none of the other characteristics of patients was associated with the marker (Figs. 1C, 2C, 3C, 4C and 5C).

**Table 3:** Association of PMS2 marker expression and the clinicopathological characteristics of patients.

			PMS2		p value
			Lost N = 3	Preserved N = 35	
Age			75.7 (5.5)	61.3 (9.5)	t:4, **p:0.025**
Gender	Male	N(%)	2(7.1)	26(92.9)	1
	Female	N(%)	1(10.0)	9(90.0)	
Diabetes	No	N(%)	1(3.1)	31(96.9)	0.059
	Yes	N(%)	2(33.3)	4(66.7)	
HTN	No	N(%)	0(0.0)	28(100)	**0.014**
	Yes	N(%)	3(30.0)	7(70.0)	
Comorbidities	No	N(%)	0(0.0)	20(100)	0.097
	Yes	N(%)	3(16.7)	15(83.3)	
Smoking	No	N(%)	1(7.1)	13(92.9)	.**040**
	Ex	N(%)	2(28.6)	5(71.4)	
	Current	N(%)	0(0.0)	16(100)	
Pathological type	Adenocarcinoma	N(%)	1(6.3)	15(93.8)	0.498
	Large cell NE carcinoma	N(%)	0(0.0)	1(100)	
	Squamous cell carcinoma	N(%)	2(18.2)	9(81.8)	
	SCLC	N(%)	0(0.0)	10(100)	
Grade	Moderate	N(%)	1(8.3)	11(91.7)	1
	Poor	N(%)	2(7.7)	24(92.3)	
Side	Right	N(%)	1(3.8)	25(96.2)	0.181
	Left	N(%)	2(20.0)	8(80.0)	
Site	Hilar	N(%)	1(6.7)	14(93.3)	0.845
	Upper and lower lobes	N(%)	0(0.0)	3(100)	
	Upper	N(%)	1(9.1)	10(90.9)	
	Lower	N(%)	1(16.7)	5(83.3)	
	Chest wall mass	N(%)	0(0.0)	1(100)	
Course	Stationary/regressive	N(%)	0(0.0)	21(100)	0.139
	Progressive	N(%)	2(15.4)	11(84.6)	
T stage	T1/2	N(%)	1(9.1)	10(90.9)	1
	T3/4	N(%)	2(7.4)	25(92.6)	
N stage	0/1	N(%)	0(0.0)	10(100)	0.552
	2/3	N(%)	3(10.7)	25(89.3)	
M stage	m0	N(%)	0(0.0)	6(100)	1
	M1A, B,C	N(%)	3(9.4)	29(90.6)	
Bone mets	No	N(%)	0(0.0)	22(100)	0.066
	Yes	N(%)	3(18.8)	13(81.2)	
Suprarenal mets	No	N(%)	0(0.0)	31(100)	**0.003**
	Yes	N(%)	3(50)	3(50)	
Stage group	II, III	N(%)	0(0.0)	5(100)	1
	IV	N(%)	3(9.1)	30(90.9)	

Bold indicates statistically significanct result where the significance level has been established at 0.05.

Table 4 shows the association of the MSH2 marker with the characteristics of the cases. HTN was significantly greater among the group with the lost marker (p-value equal 0.048). A progressive course was associated with a lost MSH2 marker (*p* = 0.048). Bone metastasis was associated with lost MSH2 marker (*p* = 0.025). Suprarenal metastasis was associated with lost MSH2 marker (*p* = 0.010). Apart from the previously mentioned parameters, none of the other characteristics of patients were associated with the marker (Figs. 1D, 2D, 3D, 4D and 5D).

**Table 4:** Association of MSH2 marker expression and clinicopathological characteristics of patients.

			MSH2		p value
			Lost N = 4	Preserved N = 34	
Age	Mean (SD)		68.5 (15)	61.8 (9.3)	t:0.9, p:0.440
Gender	Male	N(%)	2 (7.1)	26(92.9)	0.279
	Female	N(%)	2(20.0)	8(80.0)	
Diabetes	No	N(%)	2(6.3)	30(93.8)	0.110
	Yes	N(%)	2(33.3)	4(66.7)	
HTN	No	N(%)	1(3.6)	27(96.4)	**0.048**
	Yes	N(%)	3(30.0)	7(70.0)	
Comorbidities	No	N(%)	1(5.0)	19(95.0)	0.328
	Yes	N(%)	3(16.7)	15(83.3)	
Smoking	No	N(%)	2(14.3)	12(85.7)	0.090
	Ex	N(%)	2(28.6)	5(71.4)	
	Current	N(%)	0(0.0)	16(100.0)	
	Yes	N(%)	2(28.6)	5(71.4)	
Pathological type	Adenocarcinoma	N(%)	2(12.5)	14(87.5)	0.599
	Large cell NE carcinoma	N(%)	0(0.0)	1(100.0)	
	Squamous cell carcinoma	N(%)	2(18.2)	9(81.8)	
	SCLC	N(%)	0(0.0)	10(100.0)	
Grade	Moderate	N(%)	2(16.7)	10(83.3)	0.577
	Poor	N(%)	2(7.7)	24(92.3)	
Side	Right	N(%)	1(3.8)	25(96.2)	0.057
	Left	N(%)	3(30.0)	7(70.0)	
Site	Hilar	N(%)	1(6.7)	14(93.3)	0.701
	Upper and lower lobes	N(%)	0(0.0)	3(100.0)	
	Upper	N(%)	2(18.2)	9(81.8)	
	Lower	N(%)	1(16.7)	5(83.3)	
	Chest wall mass	N(%)	0(0.0)	1(100.0)	
	Yes	N(%)	0(0.0)	1(100.0)	
Course	Stationary/regressive	N(%)	0(0.0)	21(100.0)	**0.048**
	Progressive	N(%)	3(23.1)	10(76.9)	
T stage	T1/2	N(%)	2(18.2)	9(81.8)	0.564
	T3/4	N(%)	2(7.4)	25(92.6)	
N stage	0/1	N(%)	1(10.0)	9(90.0)	1
	2/3	N(%)	3(10.7)	25(89.3)	
M stage	m0	N(%)	0(0.0)	6(100.0)	1
	M1A, B,C	N(%)	4(12.5)	28(87.5)	
Bone mets	No	N(%)	0(0.0)	22(100.0)	**0.025**
	Yes	N(%)	4(25.0)	12(75.0)	
Suprarenal mets	No	N(%)	1(3.2)	30(96.8)	**0.010**
	Yes	N(%)	3(50.0)	3(50.0)	
Stage group	II, III	N(%)	0(0.0)	5(100.0)	1
	IV	N(%)	4(12.1)	29(87.9)	

Bold indicates statistically significanct result where the significance level has been established at 0.05.

Table 5 shows the association of the MSH6 marker with characteristics of cases. HTN was significantly higher among the group with the lost marker (p-value equal 0.048). A progressive course was correlated with lost MSH6 marker (*p* = 0.048). Bone metastasis was correlated with lost MSH6 marker (*p* = 0.025). Suprarenal metastasis was associated with lost MSH6 marker (*p* = 0.010). Apart from the previously mentioned parameters, However, the marker was not associated with any of the other patient characteristics (Figs. 1E, 2E, 3E, 4E and 5E).

**Table 5:** Association of MSH6 marker expression and clinicopathological characteristics of patients.

			MSH6		p value
			Lost N = 4	Preserved N = 34	
Age	Mean (sd)		68.5 (15)	61.8 (9.3)	T:0.9, p: 0.44
Gender	Male	N(%)	2(7.1)	26(92.9)	0.279
	Female	N(%)	2(20.0)	8(80.0)	
Diabetes	No	N(%)	2(6.3)	30(93.8)	0.110
	Yes	N(%)	2(33.3)	4(66.7)	
HTN	No	N(%)	1(3.6)	27(96.4)	**0.048**
	Yes	N(%)	3(30.0)	7(70.0)	
Comorbidities	No	N(%)	1(5.0)	19(95.0)	0.328
	Yes	N(%)	3(16.7)	15(83.3)	
Smoking	No	N(%)	2(14.3)	12(85.7)	0.088
	Ex	N(%)	2(28.6)	5(71.4)	
	Current	N(%)	0(0.0)	16(100.0)	
	Yes	N(%)	2(28.6)	5(71.4)	
Pathological type	Adenocarcinoma	N(%)	2(12.5)	14(87.5)	0.594
	Large cell NE carcinoma	N(%)	0(0.0)	1(100.0)	
	Squamous cell carcinoma	N(%)	2(18.2)	9(81.8)	
	SCLC	N(%)	0(0.0)	10(100.0)	
Side	Right	N(%)	1(3.8)	25(96.2)	0.057
	Left	N(%)	3(30.0)	7(70.0)	
Site	Hilar	N(%)	1(6.7)	14(93.3)	0.698
	Upper and lower lobes	N(%)	0(0.0)	3(100.0)	
	Upper	N(%)	2(18.2)	9(81.8)	
	Lower	N(%)	1(16.7)	5(83.3)	
	Chest wall mass	N(%)	0(0.0)	1(100.0)	
Course	Stationary/regressive	N(%)	0(0.0)	21(100.0)	**0.048**
	Progressive	N(%)	3(23.1)	10(76.9)	
T stage	T1/2	N(%)	2 (18.2 )	9(81.8)	0.564
	T3/4	N(%)	2(7.4)	25(92.6)	
N stage	0/1	N(%)	1 (10.0 )	9(90.0)	1
	2/3	N(%)	3(10.7)	25(89.3)	
M stage	m0	N(%)	0(0.0)	6(100.0)	1
	M1A, B,C	N(%)	4(12.5)	28(87.5)	
Bone mets	No	N(%)	0(0.0)	22(100.0)	**0.025**
	Yes	N(%)	4(25.0)	12(75.0)	
Suprarenal mets	No	N(%)	1(3.2)	30(96.8)	**0.010**
	Yes	N(%)	3(50.0)	3(50.0)	
Stage group	II, III	N(%)	0(0.0)	5(100.0)	1
	IV	N(%)	4(12.1)	29(87.9)	

Bold indicates statistically significanct result where the significance level has been established at 0.05.

### MMR genes expression in relation to each other’s

Table 6 shows correlations between the four markers. All the markers were significantly correlated (strong correlation) with each other, with perfect correlation between MLH & PMS2 markers and perfect correlation between MSH6, & MSH2 markers.

**Table 6:** Correlation between markers to each other’s.

			PMS2	MSH2	MSH6
Spearman’s rho	MLH1	Correlation coefficient	1.000^**^	0.854^**^	0.854^**^
		Sig. (2-tailed)		0.000	0.000
	PMS2	Correlation coefficient		0.854^**^	0.854^**^
		Sig. (2-tailed)		0.000	0.000
	MSH2	Correlation coefficient			1.000^**^
		Sig. (2-tailed)			

**Correlation is significant at the 0.01 level (2-tailed).

### Association between MMR genes expression and survival

Table 7 shows the cases’ overall survival (OS) in relation to their marker groups. It shows that the median overall survival among cases with lost markers was significantly lower than patients with preserved markers, with p value < 0.05 for all markers.

**Table 7:** Association between all markers and overall survival.

		Total N	Events N	Censored N (%)	Survival time median (SE)	X^2*^	p
MLH1	Lost	3	2	1 (33.3)	3 (0.0)	34.6	**≤ 0.001**
	Preserved	34	3	31(91.2)	36 (18.5)		
PMS2	Lost	3	2	1 (33.3)	3 (0.0)	34.6	**≤ 0.001**
	Preserved	34	3	31(91.2)	36 (18.5)		
MSH2	Lost	4	2	2 (50)	3 (1.5)	16.6	**≤ 0.001**
	Preserved	33	3	30 (90.9)	36 (18.5)		
MSH6	Lost	4	2	2 (50)	3 (1.5)	16.6	**≤ 0.001**
	Preserved	33	3	30 (90.9)	36 (18.5)		

Bold indicates statistically significanct result where the significance level has been established at 0.05.

*Log rank (Mantel-Cox).

## Discussion

The present retrospective study included 38 LC patients; their mean age ± SD was 62.5 ± 10 years, and the majority of them were males (74%). Similar cases characteristics were reported in a research conducted on Egyptian LC patients in another institute study^4^. Our findings are also comparable to those of the global cohort (63 years) and Middle East and Africa cohort (61years)^16,17^.

The two main histopathologic forms of LC are SCLC& NSCLC. NSCLC includes, adenocarcinoma, squamous cell carcinoma and others^8,18^. The precise differentiation among special subtypes of NSCLC before starting therapy is fundamental, as each subtype exhibits a different prognosis. Histological subtypes of NSCLC have various histologic bases, exclusive genetic profiles, and contrasting clinical presentations^19^.

In this context, our histopathological findings showed that the majority (73.7%) of studied patients had NSCLC versus (26.3%) with SCLC. Adenocarcinoma was the predominant variant (43.1%) of all NSCLC tumors. Also, 68.4% of our included LC cases were poorly differentiated. In addition, 86.8% of patients were diagnosed as advanced (stage IV), and 65.8% had stage IVB.

Our patients` histopathological characteristics are compatible with the.

(2021) WHO classification of thoracic tumors. Similar histopathological characteristics and frequencies were reported in older studies^20^. Our findings are also consistent with previous studies which mentioned that NSCLC cases mostly exhibit advanced disease stages at the time of diagnosis, especially in developing countries; consequently, they are characterized by a poor prognosis^6,21^. As regards Egypt, a study reported that the majority of Egyptian cases diagnosed with LC exhibit either metastatic or locally advanced disease^22^.

The onset of lung carcinogenesis is implied by cellular and molecular components of the tumor microenvironment (TME). Both exogenous factors including tobacco smoke, and endogenous factors, such as dMMR in addition to dysfunctions of homologous recombination (HR) and base excision repair (BER) can result in further DNA damage and, as a result, more mutations could accumulate in tumor cells and contribute to lung multistage carcinogenesis^23^.

Regarding smoking as the main exogenous risk factor with mutagenic influence involved in lung carcinogenesis, in the current study, the smoking history at diagnosis of 37 patients was as follows: 37.8% were non-smokers while 62.2% were smokers. Smokers were divided into two sub-groups: one included 43.2% current smokers at diagnosis time and the other included 18.9% ex-smokers at diagnosis time.

LC results from the final stage of multistage modal carcinogenesis with gradually cumulative epigenetic & genetic alterations^24^. Smoking remains the most significant and well-established extrinsic contributor correlated to the development of LC^25^. Notably, smoking is more strongly associated with LC forms such as squamous cell carcinoma (SCC) and small cell lung cancer (SCLC) than adenocarcinoma & large-cell carcinoma^26^. In Egypt, the occurrence of LC is significantly affected by tobacco consumption, with an age-standardized occurrence of 43.4%, especially among males, which exceeds the global average. The mechanism was proposed to be through DNA damage, chronic growth factors, oxidative stress, inflammation activity, DNA repair dysfunction, and elevated cytokines^27^.

DNA MMR is essential for preserving genome stability by recognizing & repairing single-based mismatch defects, involving deletions, mis-incorporations, and insertions, that arise throughout DNA replication^28^. The integrity of DNA mismatch repair is often assessed clinically via immunohistochemistry labeling for the expression of the four mismatch repair proteins. The predominant dysfunctions identified in cancers exhibiting DNA mismatch repair deficit is the lack of MutL homolog 1/Postmeiotic segregation enhanced two (MLH1/PMS2) and/or MutL homolog 2/MutL homolog six expression(MSH2/MSH6)^28^.

The hypermutator phenotype of microsatellite instability (MSI-H) is generally attributed to mutations in the mismatch repair genes (MLH1, PMS2, MSH2 & MSH6) in malignancies exhibiting poor mismatch repair mechanisms. The accumulation of somatic mutations & the production of “non-self” neoantigens are consequences of MSI-H/dMMR. This situation subsequently stimulates the host’s anti-tumor immune response. MSI-H/dMMR was widely identified & characterized in malignancies related to Lynch syndrome, including gastrointestinal adenocarcinoma, endometrial cancer & colorectal cancer. Nevertheless, they are infrequently observed in breast cancer, lung adenocarcinoma, or prostate cancer^29^.

Regarding our aim to evaluate whether lung tumors have dMMR, immunohistochemistry testing was chosen as an accurate, easy, and common method to examine the expression of mismatch repair proteins (MLH1, PMS2, MSH 2 & MSH6). In lung malignancies. If the expression of all 4 proteins is present, the tumor is classified as pMMR, while dMMR is the case with absent one proteinat least^30^.

Only 7.8% of patients had lost MLH1 and PMS2 markers, while 10.5% of them had lost MSH2 and MSH6 markers. These results are similar to previous reseach demonstrating that MLH1, MSH6, and PMS2 expression losses were 8.8, 5.9, and 20.6%, respectively. According to these results, only seven tumors (20.6%) have been identified as microsatellite unstable (MSI)^31^.

These outcomes are also consistent with another study that demonstrated diminished expression levels of the mutl homolog 1 (MLH1) and mutl homolog 2 (MSH2) proteins in hereditary non polyposis cancer colon (HNPCC) and also other human malignancies, such as LC. In addition to the germline mutations that have been identified in the genes MLH1 and MSH2, numerous polymorphisms have also been identified^32^.

As regards MMR gene expression in relation to other clinicopathological parameters, age was significantly greater among the group with lost MLH1 and PMS2 markers. Also, HTN was significantly greater among the group with both markers lost. As regards MSH2 and MSH6, HTN was significantly higher among the group losing both markers. In colorectal carcinoma, The microsatellite stability status was statistically significantly correlated with the age of cases, tumor site, and grade^33^.

On the contrary, according to research done by Ye et al., the risk of MMR genes mutation was not elevated by age. Age was not significantly correlated with any MMR genes in the lung cancer group, as indicated by the correlation analysis. Within the control group, the correlation analysis among age & MMR genes demonstrated that 5 MMR genes have been relatively weakly related to age^34^.

Our findings indicated that smoking was significantly correlated with expression of MLH1 and PMS2 markers (*P* = 0.040) (Tables 2 and 3). This finding is in accordance with the outcomes of a study done by Li et al. (2013), who found that nonsmokers exhibited a higher rate of hMLH1 expression than smokers. Also, adenocarcinoma exhibited a higher frequency of expression than squamous cell carcinoma. However, the expression of hMLH1 did not differ significantly among squamous cell carcinoma & adenocarcinoma when the influence of smoking history has been controlled. Conversely, a significant difference has been observed when the pathological classification has been controlled. This suggests that smoking history, rather than pathological classification, is the primary factor influencing hMLH1 expression^35^.

In previous research, Xinarianos et al. also demonstrated that heavy smokers exhibited a higher rate of lower hMLH1 expression. Moreover, the genetic instability attributed to the decreased expression of the hMLH1 protein & hMSH2 protein is correlated to smoking status^36^. In another research, a comprehensive clinic-genomic landscape of MSI-H lung cancers was presented, revealing that MSI-H defines a rare subset of LC that are correlated with high tumor mutational burden, smoking, & MLH1 inactivation^37^.

As regards other clinicopathological characteristics, we didn’t find a significant correlation between MMR genes expression and the histologic tumor type of LC. However, the results of Kanellis et al. study suggested that loss of MSH2 expression is common in NSCLC. Additionally, they suggested that the expression of MSH2 could be assessed for the purpose of screening of cytological material obtained from fine needle aspirations of the lung^38^. Similar to our results, Akcay et al. study documented no relationship between MSI and pathologic and clinical stages of lung adenocarcinomas^31^.

As regards the correlation between lost MMR genes and distant metastasis. Suprarenal metastasis was associated with lost MLH1 and PMS2 markers. Both bone and suprarenal metastasis were associated with lost MSH2 and MSH6 markers as well. Chen et al. (2024) conducted a study that was similar to ours. They found that cerebral metastases in lung cancer are influenced by somatic alterations of PMS2. They also suggested the potential of PMS2 targeting for therapeutic intervention in life-threatening brain metastases^39^.

In addition, endometrial carcinoma with MMR-deficiency exhibited a high rate of recurrence or metastasis. Transcriptional analysis revealed a higher risk of migration & metastasis indicating that clonal mismatch repair deficiency may be a contributing factor to tumor aggressiveness^40^. But the Akcay et al. study documented no correlation between MSI and the presence of lympho-vascular invasion, lymph node metastases & distant metastases^31^.

We also found correlations between markers. All the markers were significantly correlated (strong correlation) to each other, with perfect correlations between MSH6, & MSH2, and between MLH and PMS2. Our findings are consistent with numerous previous sudies. Some authors documented that those alterations in MSH2 would be expected to cause a loss of staining of both proteins, MSH2 and MSH6^40^. Another study found that cyclin dependent kinase2 (CDK2) phosphorylates PMS2 and MLH1 in vitro & phosphorylation of PMS2 influences its interaction with MLH1^41^. Also, Li et al. (2013) found that MSH2 expression was significantly correlated to the MLH1 expression^35^.

The Mutl alpha complex is triggered upon the detection of DNA mismatches, insertions, or deletions by MutS alpha & MutS beta heterodimers, wherein the PMS2 gene (located on chromosome seven) encodes an endonuclease that forms a heterodimer with MLH1. Thus, PMS2 is unstable in the absence of MLH1 and the loss of MLH1 expression as a result of mutations typically results in the loss of PMS2 expression as well^42^.

Additionally, the MSH6 gene (on chromosome 2) encodes a component of the Muts family of proteins that are involved in DNA MMR. This protein heterodimerizes with MSH2 to form the Muts alpha complex, which functions as a bidirectional molecular switch for the mismatch repair machinery. So, the expression of MSH6 protein is unstable in the absence of MSH2, and the loss of MSH2 expression as a consequence of mutations typically leads to the loss of MSH6 expression^42^. Mutations in MSH2 are typically the cause of negative staining of MSH6, which should therefore raise concern about germline mutations^43^.

Regarding association between MMR genes expression and prognosis. Progressive course has been correlated to lost expression of both markers MSH2 and MSH6 (P-value equal 0.048) (Tables 4 and 5). Also, we found that the median overall survival between cases with lost markers was significantly lower than that of cases with preserved markers, with a p value < 0.05 for all markers (Table 7). The survival analysis of forty-three differentially expressed MMRgenes in LC revealed that three differentially expressed MMR genes had survival significance, which is consistent with the findings of this research^34^.

Also, our results are in agreement with recent research in colorectal carcinoma. They demonstrated that the recurrence free survival (RFS) rate of deficient mismatch repair colorectal cases is lower than that of proficient mismatch repair^44^. In another study, the prognostic & predictive value of deficient mismatch repair/MSI-H in colorectal has been evidenced^45^.

In comparison to the Micro satellite stable (MSS) group, cases with microsatellite instability (MSI) group also appeared to have significantly lower overall survival rates, as discovered by other authors. This held true in multivariable analysis, as microsatellite instability status has been independently correlated to worse overall survival^46^. One potential explanation is that cases with malignancies of high MSI may experience early disease recurrence. This phenomenon has been previously described by Matteo et al.^47^.

But our result disagrees with the results shown by previous searchers who stated that the disease-free survival (DFS) and overall survival (OS) of the cases were not influenced by the expression status of MMR proteins^36^. Moreover, in another study, no prognostic relationship was identified between MSI and overall survival^31^.

In order to treat tumors more effectively, molecular targeting of drugs is beginning to become more important. Targeted therapies are being utilized more frequently to improve the clinical outcomes of cases with NSCLC, with encouraging results, especially for cases with specific molecular features^48^. The essential roles of the mismatch repair pattern of genes in the development of tumor immune microenvironment in lung adenocarcinoma cases were demonstrated by identification of a distinct mismatch repair pattern of modification regulation pattern & their related various immune characters, sensitivity of neoadjuvant chemotherapy & prognoses^49^.

Keeping in mind the steady and continuous advancing cancer treatment and novel biomarkers in LC; The four proteins, mutl homolog 2, mutl homolog 1, mutl homolog 6, and postmitotic segregating increased 2, should be evaluated by immunohistochemistry (IHC). This is strongly encouraged. It may be broadly introduced at individual medical institutions as prognostic markers for LC particularly after the approval of an in vitro diagnostic kit for detecting dMMR function in Japan in December 2021^30^.

Additional research on larger sample sizes is mandatory to evaluate the dMMR lung tumors statuses and their role regarding different treatment modalities particularly immune therapy & response to immune check point blockade. Other causes of dMMR are not studied here and it is important to get a complete picture of dMMR as MMR protein loss may imply suboptimal mismatch repair status of the tumor. Although MSI analysis is not mandatory, it may be complementary to assess instability as several studies reported microsatellite stability despite the loss of MMR proteins detected by IHC.

Our study has many points of strengths; IHC offers a superior advantage over MSI testing in that it can identify genes that are responsible for diffident mismatch repair status by analyzing the expression pattern of proteins that are absent. Fortunately, we were able to assess the expression of all 4 mismatch repair proteins PMS2, MLH1, MSH2 & MSH6 in their entirety. Although sample size is smaller than similar studies in different geographical areas, our results regarding MMR protein expression are relevant and comparable to that of larger studies indicating that the high incidence of dMMR may be evident in our LC population. The only limitation of our study was small sample size as our study represent a single center retrospective study.

## Conclusions

The current study recommends evaluation of the four proteins, MSH2, MLH1, PMS2 and MSH6 as biomarkers which could guide LC therapy. Also, our results may be valuable to elucidate the pathophysiology of smoking related LC, a crucial cause of sustainable global LC burden. Moreover, in-depth biological studies are imperative to elucidate the precise roles and mechanisms of these identified markers. This will advance our management strategies for LC cases and even guide immune checkpoint inhibitor therapy for LC as well as other solid malignancies.

## Electronic Supplementary Material

Below is the link to the electronic supplementary material.

Supplementary Material 1

## Data Availability

The datasets used and/or analyzed during the current study available from the corresponding author on reasonable request.

## Abbreviations

BER: Base excision repair
CDK2: Cyclin dependent kinase2
DM: Diabetes mellitus
dMMR: Deficient mismatch repair
DSBR: Double-strand break repair
HNPCC: Hereditary non polyposis cancer colon
HR: Homologous recombination
HTN: Hypertension
IHC: Immunohistochemistry
LC: Lung cancer
MLH1: Mutl homolog 1
MMR: Mismatch repair
MSH2: Mutl homolog 2
MSH6: Mutl homolog 6
MSI: Microsatellite instability
MSI-H: The hypermutator phenotype of MSI
MSS: Micro satellite stable
NER: Nucleotide excision repair
NSCLC: Non-small cell lung cancer
OS: Overall survival
PFS: Progression-free survival
pMMR: Proficient mismatch repair
PMS2: Postmeiotic segregation increased 2
RFS: Recurrence free survival
SCC: Squamous cell carcinoma
SCLC: Small cell lung cancer
TME: Tumor microenvironment
