# Supplementary material for: DNA mismatch repair (MMR) genes expression in lung cancer and its correlation with different clinicopathologic parameters
Source: Sci Rep. 2025 Jan 6;15:885. doi: 10.1038/s41598-024-83067-2 (PMC11704133; doi:10.1038/s41598-024-83067-2)
Supplement: Supplementary file 1 — Supplementary Material 1 [file 41598_2024_83067_MOESM1_ESM.docx]

Supplementary Table 2: Association of MLH1 marker expression and the clinicopathological characteristics of patients:

|  |  |  | MLH1 | |  |
| --- | --- | --- | --- | --- | --- |
|  |  |  |  |  |  |
|  |  |  | Lost  N=3 | Preserved  N=35 | p value |
| Age | Mean (SD) |  | 75.7 (5.5) | 61.3(9.4) | t:2.6, p:0.015 |
| Gender | male | N% | 2(7.1%) | 26(92.9%) | 1 |
|  | female | N% | 1(10.0%) | 9(90.0%) |  |
| Diabetes | no | N(%) | 1(3.1%) | 31(96.9%) | 0.059 |
|  | yes | N(%) | 2(33.3%) | 4(66.7%) |  |
| HTN | no | N(%) | 0(0.0%) | 28(100.0%) | 0.014 |
|  | yes | N(%) | 3(30.0%) | 7(70.0%) |  |
| Comorbidities | no | N(%) | 0(0.0) | 20(100.0) | 0.097 |
|  | yes | N(%) | 3(16.7) | 15(83.3) |  |
| Smoking | no | N(%) | 1(7.1) | 13(92.9) | .045^b^ |
|  | ex | N(%) | 2(28.6) | 5(71.4) |  |
|  | current | N(%) | 0(0.0) | 16(100.0) |  |
| symptoms. cough | no | N(%) | 1(9.1) | 10(90.9) | 1 |
|  | yes | N(%) | 2(7.4) | 25(92.6) |  |
| chest.pain | no | N(%) | 3(11.1) | 24(88.9) | 0.542 |
|  | yes | N(%) | 0(0.0) | 11(100.0) |  |
| symp.haemoptysis | no | N(%) | 2(6.7) | 28(93.3) | 0.519 |
|  | yes | N(%) | 1(12.5) | 7(87.5) |  |
| Symp.shortness.of.breath | no | N(%) | 2(12.5) | 14(87.5) | 0.562 |
|  | yes | N(%) | 1(4.5) | 21(95.5) |  |
| sympt.pathologi.fracture | no | N(%) | 2(6.5) | 29(93.5) | 0.467 |
|  | yes | N(%) | 1(14.3) | 6(85.7) |  |
| pathological.type | adenocarcinoma | N(%) | 1(6.3) | 15(93.8) | .489 |
|  | large cell neuroendocrine carcinoma | N(%) | 0(0.0) | 1(100) |  |
|  | squamous cell carcinoma | N(%) | 2(18.2) | 9(81.8) |  |
|  | SCLC | N(%) | 0(0.0) | 10(100) |  |
| grade | moderate | N(%) | 1(8.3) | 11(91.7) | 1 |
|  | poor | N(%) | 2(7.7) | 24(92.3) |  |
| side | right | N(%) | 1(3.8) | 25(96.2) | 0.181 |
|  | left | N(%) | 2(20.0) | 8(80.0) |  |
| site | hilar | N(%) | 1(6.7) | 14(93.3) | .832 |
|  | upper and lower lobes | N(%) | 0(0.0) | 3(100) |  |
|  | upper | N(%) | 1(9.1) | 10(90.9) |  |
|  | lower | N(%) | 1(16.7) | 5(83.3) |  |
|  | chest wall mass | N(%) | 0(0.0) | 1(100) |  |
| ct.pleural.effusion | no | N(%) | 3(14.3) | 18(85.7) | 0.238 |
|  | yes | N(%) | 0(0.0) | 17(100) |  |
| ct.lymphnodes | no | N(%) | 0(0.0) | 9(100) | 1 |
|  | yes | N(%) | 3(10.3) | 26(89.7) |  |
| pulmonary.metastatic.nodules | no | N(%) | 1(4.8) | 20(95.2) | 0.577 |
|  | yes | N(%) | 2(11.8) | 15(88.2) |  |
| collapse | no | N(%) | 3(10.3) | 26(89.7) | 1 |
|  | yes | N(%) | 0(0.0) | 9(100) |  |
| ct.chestwall.mass | no | N(%) | 3(9.1) | 30(90.9) | 1 |
|  | yes | N(%) | 0(0.0) | 5(100) |  |
| bone.metas | no | N(%) | 0(0.0) | 22(100) | 0.066 |
|  | yes | N(%) | 3(18.8) | 13(81.2) |  |
| brain.mets | no | N(%) | 3(9.1) | 30(90.9) | 1 |
|  | yes | N(%) | 0(0.0) | 5(100) |  |
| peripheral.ln | no | N(%) | 3(9.4) | 29(90.6) | 1 |
|  | yes | N(%) | 0(0.0) | 6(100) |  |
| liver.metastasis | no | N(%) | 2(7.4) | 25(92.6) | 1 |
|  | yes | N(%) | 1(9.1) | 10(90.9) |  |
| suprarenal.mets | no | N(%) | 0(0.0) | 31(100) | 0.003 |
|  | yes | N(%) | 3(50) | 3(50) |  |
| surgery | no | N(%) | 3(8.3) | 33(91.7) | 1 |
|  | yes | N(%) | 0(0.0) | 1(100) |  |
| course.group | stationary/regressive | N(%) | 0(0.0) | 21(0.0) | 0.139 |
|  | progressive | N(%) | 2(15.4) | 11(84.6) |  |
| n.gp | 0/1 | N(%) | 0(0.0) | 10(100) | 0.552 |
|  | 2/3 | N(%) | 3(10.7) | 25(89.3) |  |
| T | T1/2 | N(%) | 1(9.1) | 10(90.9) | 1 |
|  | T3/4 | N(%) | 2(7.4) | 25(92.6) |  |
| M | m0 | N(%) | 0(0.0) | 6(100) | 1 |
|  | M1A,B,C | N(%) | 3(9.4) | 29(90.6) |  |
| stage.gp | II,III | N(%) | 0(0.0) | 5(100) | 1 |
|  | IV | N(%) | 3(9.1) | 30(90.9) |  |

Supplementary Table 3: Association of PMS2 marker expression and the clinicopathological characteristics of patients:

|  |  |  | PMS2 | |  |
| --- | --- | --- | --- | --- | --- |
|  |  |  | Lost  N=3 | Preserved  N=35 |  |
| Age |  |  | 75.7 (5.5) | 61.3 (9.5) | t:4, p:0.025 |
| gender | male | N(%) | 2(7.1) | 26(92.9) | 1 |
|  | female | N(%) | 1(10.0) | 9(90.0) |  |
| diabetes | no | N(%) | 1(3.1) | 31(96.9) | 0.059 |
|  | yes | N(%) | 2(33.3) | 4(66.7) |  |
| HTN | no | N(%) | 0(0.0) | 28(100) | 0.014 |
|  | yes | N(%) | 3(30.0) | 7(70.0) |  |
| comorbidities | no | N(%) | 0(0.0) | 20(100) | 0.097 |
|  | yes | N(%) | 3(16.7) | 15(83.3) |  |
| smoking | no | N(%) | 1(7.1) | 13(92.9) | .040 |
|  | ex | N(%) | 2(28.6) | 5(71.4) |  |
|  | current | N(%) | 0(0.0) | 16(100) |  |
| symptoms.1.cough | no | N(%) | 1(9.1) | 10(90.9) | 1 |
|  | yes | N(%) | 2(7.4) | 25(92.6) |  |
| sym.chest.pain | no | N(%) | 3(11.1) | 24(88.9) | 0.542 |
|  | yes | N(%) | 0(0.0) | 11(100) |  |
| symp.haemoptysis | no | N(%) | 2(6.7) | 28(93.3) | 0.519 |
|  | yes | N(%) | 1(12.5) | 7(87.5) |  |
| Symp.shortness.of.breath | no | N(%) | 2(12.5) | 14(87.5) | 0.562 |
|  | yes | N(%) | 1(4.5) | 21(95.5) |  |
| sympt.pathologi.fracture | no | N(%) | 2(6.5) | 29(93.5) | 0.467 |
|  | yes | N(%) | 1(14.3) | 6(85.7) |  |
| pathological.type | adenocarcinoma | N(%) | 1(6.3) | 15(93.8) | .498 |
|  | large cell neuroendocrine carcinoma | N(%) | 0(0.0) | 1(100) |  |
|  | squamous cell carcinoma | N(%) | 2(18.2) | 9(81.8) |  |
|  | SCLC | N(%) | 0(0.0) | 10(100) |  |
| grade | moderate | N(%) | 1(8.3) | 11(91.7) | 1 |
|  | poor | N(%) | 2(7.7) | 24(92.3) |  |
| side | right | N(%) | 1(3.8) | 25(96.2) | 0.181 |
|  | left | N(%) | 2(20.0) | 8(80.0) |  |
| site | hilar | N(%) | 1(6.7) | 14(93.3) | .845 |
|  | upper and lower lobes | N(%) | 0(0.0) | 3(100) |  |
|  | upper | N(%) | 1(9.1) | 10(90.9) |  |
|  | lower | N(%) | 1(16.7) | 5(83.3) |  |
|  | chest wall mass | N(%) | 0(0.0) | 1(100) |  |
| ct.pleural.effusion | no | N(%) | 3(14.3) | 18(85.7) | 0.238 |
|  | yes | N(%) | 0(0.0) | 17(100) |  |
| ct.lymphnodes | no | N(%) | 0(0.0) | 9(100) | 1 |
|  | yes | N(%) | 3(10.3) | 26(89.7) |  |
| pulmonary.metastatic.nodules | no | N(%) | 1(4.8) | 20(95.2) | 0.577 |
|  | yes | N(%) | 2(11.8) | 15(88.2) |  |
| collapse | no | N(%) | 3(10.3) | 26(89.7) | 1 |
|  | yes | N(%) | 0(0.0) | 9(100) |  |
| ct.chestwall.mass | no | N(%) | 3(9.1) | 30(90.9) | 1 |
|  | yes | N(%) | 0(0.0) | 5(100) |  |
| bone.metas | no | N(%) | 0(0.0) | 22(100) | 0.066 |
|  | yes | N(%) | 3(18.8) | 13(81.3) |  |
| brain.mets | no | N(%) | 3(9.1) | 30(90.9) | 1 |
|  | yes | N(%) | 0(0.0) | 5(100) |  |
| peripheral.ln | no | N(%) | 3(9.4) | 29(90.6) | 1 |
|  | yes | N(%) | 0(0.0) | 6(100) |  |
| liver.metastasis | no | N(%) | 2(7.4) | 25(92.6) | 1 |
|  | yes | N(%) | 1(9.1) | 10(90.9) |  |
| suprarenal.mets | no | N(%) | 0(0.0) | 31(100) | 0.003 |
|  | yes | N(%) | 3(50) | 3(50) |  |
| surgery | no | N(%) | 3(8.3) | 33(91.7) | 1 |
|  | yes | N(%) | 0(0.0) | 1(100) |  |
| course.group | stationary/regressive | N(%) | 0(0.0) | 21(100) | 0.139 |
|  | progressive | N(%) | 2(15.4) | 11(84.6) |  |
| t.gp | T1/2 | N(%) | 1(9.1) | 10(90.9) | 1 |
|  | T3/4 | N(%) | 2(7.4) | 25(92.6) |  |
| n.gp | 0/1 | N(%) | 0(0.0) | 10(100) | 0.552 |
|  | 2/3 | N(%) | 3(10.7) | 25(89.3) |  |
| M.GP | m0 | N(%) | 0(0.0) | 6(100) | 1 |
|  | M1A,B,C | N(%) | 3(9.4) | 29(90.6) |  |
| stage.gp | II,III | N(%) | 0(0.0) | 5(100) | 1 |
|  | IV | N(%) | 3(9.1) | 30(90.9) |  |

Supplementary Table 4: Association of MSH2 marker expression and the clinicopathological characteristics of patients:

|  |  |  | MSH2 | |  |
| --- | --- | --- | --- | --- | --- |
|  |  |  | Lost  N=4 | Preserved  N=34 |  |
| Age | Mean (SD) |  | 68.5 (15) | 61.8 (9.3) | t:0.9, p:0.440 |
| gender | male | N(%) | 2 (7.1) | 26(92.9) | 0.279 |
|  | female | N(%) | 2(20.0) | 8(80.0) |  |
| diabetes | no | N(%) | 2(6.3) | 30(93.8) | 0.110 |
|  | yes | N(%) | 2(33.3) | 4(66.7) |  |
| HTN | no | N(%) | 1(3.6) | 27(96.4) | 0.048 |
|  | yes | N(%) | 3(30.0) | 7(70.0) |  |
| comorbidities | no | N(%) | 1(5.0) | 19(95.0) | 0.328 |
|  | yes | N(%) | 3(16.7) | 15(83.3) |  |
| smoking | no | N(%) | 2(14.3) | 12(85.7) | .090 |
|  | ex | N(%) | 2(28.6) | 5(71.4) |  |
|  | current | N(%) | 0(0.0) | 16(100.0) |  |
| symptoms.1.cough | no | N(%) | 1(9.1) | 10(90.9) | 1 |
|  | yes | N(%) | 3(11.1) | 24(88.9) |  |
| sym.chest.pain | no | N(%) | 3(11.1) | 24(88.9) | 1 |
|  | yes | N(%) | 1(9.1) | 10(90.9) |  |
| symp.haemoptysis | no | N(%) | 3(10.0) | 27(90.0) | 1 |
|  | yes | N(%) | 1(12.5) | 7(87.5) |  |
| Symp.shortness.of.breath | no | N(%) | 2(12.5) | 14(87.5) | 1 |
|  | yes | N(%) | 2(9.1) | 20(90.9) |  |
| sympt.pathologi.fracture | no | N(%) | 2(6.5) | 29(93.5) | 0.147 |
|  | yes | N(%) | 2(28.6) | 5(71.4) |  |
| pathological.type | adenocarcinoma | N(%) | 2(12.5) | 14(87.5) | .599 |
|  | large cell neuroendocrine carcinoma | N(%) | 0(0.0) | 1(100.0) |  |
|  | squamous cell carcinoma | N(%) | 2(18.2) | 9(81.8) |  |
|  | SCLC | N(%) | 0(0.0) | 10(100.0) |  |
| grade | moderate | N(%) | 2(16.7) | 10(83.3) | 0.577 |
|  | poor | N(%) | 2(7.7) | 24(92.3) |  |
| side | right | N(%) | 1(3.8) | 25(96.2) | 0.057 |
|  | left | N(%) | 3(30.0) | 7(70.0) |  |
| site | hilar | N(%) | 1(6.7) | 14(93.3) | .701 |
|  | upper and lower lobes | N(%) | 0(0.0) | 3(100.0) |  |
|  | upper | N(%) | 2(18.2) | 9(81.8) |  |
|  | lower | N(%) | 1(16.7) | 5(83.3) |  |
|  | chest wall mass | N(%) | 0(0.0) | 1(100.0) |  |
| ct.pleural.effusion | no | N(%) | 3(14.3) | 18(85.7) | 0.613 |
|  | yes | N(%) | 1(5.9) | 16(94.1) |  |
| ct.lymphnodes | no | N(%) | 1(11.1) | 8(88.9) | 1 |
|  | yes | N(%) | 3(10.3) | 26(89.7) |  |
| pulmonary.metastatic.nodules | no | N(%) | 1(4.8) | 20(95.2) | 0.307 |
|  | yes | N(%) | 3(17.6) | 14(82.4) |  |
| collapse | no | N(%) | 4(13.8) | 25(86.2) | 0.554 |
|  | yes | N(%) | 0(0.0) | 9(100.0) |  |
| ct.chest wall.mass | no | N(%) | 4(12.1) | 29(87.9) | 1 |
|  | yes | N(%) | 0(0.0) | 5(100.0) |  |
| bone.metas | no | N(%) | 0(0.0) | 22(100.0) | 0.025 |
|  | yes | N(%) | 4(25.0) | 12(75.0) |  |
| brain.mets | no | N(%) | 4(12.1) | 29(87.9) | 1 |
|  | yes | N(%) | 0(0.0) | 5(100.0) |  |
| peripheral.ln | no | N(%) | 4(12.5) | 28(87.5) | 1 |
|  | yes | N(%) | 0(0.0) | 6(100.0) |  |
| liver.metastasis | no | N(%) | 3(11.1) | 24(88.9) | 1 |
|  | yes | N(%) | 1(9.1) | 10(90.9) |  |
| suprarenal.mets | no | N(%) | 1(3.2) | 30(96.8) | 0.010 |
|  | yes | N(%) | 3(50.0) | 3(50.0) |  |
| surgery | no | N(%) | 4(11.1) | 32(88.9) | 1 |
|  | yes | N(%) | 0(0.0) | 1(100.0) |  |
| course.group | stationary/regressive | N(%) | 0(0.0) | 21(100.0) | 0.048 |
|  | progressive | N(%) | 3(23.1) | 10(76.9) |  |
| t.gp | T1/2 | N(%) | 2(18.2) | 9(81.8) | 0.564 |
|  | T3/4 | N(%) | 2(7.4) | 25(92.6) |  |
| n.gp | 0/1 | N(%) | 1(10.0) | 9(90.0) | 1 |
|  | 2/3 | N(%) | 3(10.7) | 25(89.3) |  |
| M.GP | m0 | N(%) | 0(0.0) | 6(100.0) | 1 |
|  | M1A,B,C | N(%) | 4(12.5) | 28(87.5) |  |
| stage.gp | II,III | N(%) | 0(0.0) | 5(100.0) | 1 |
|  | IV | N(%) | 4(12.1) | 29(87.9) |  |

Supplementary Table 5: Association of MSH6 marker expression and the clinicopathological characteristics of patients:

|  |  |  | MSH6 | | p value |
| --- | --- | --- | --- | --- | --- |
|  |  |  | Lost  N=4 | Preserved  N=34 |  |
| Age | Mean (sd) |  | 68.5 (15) | 61.8 (9.3) | T:0.9, p: 0.44 |
| gender | male | N(%) | 2(7.1) | 26(92.9) | 0.279 |
|  | female | N(%) | 2(20.0) | 8(80.0) |  |
| diabetes | no | N(%) | 2(6.3) | 30(93.8) | 0.110 |
|  | yes | N(%) | 2(33.3) | 4(66.7) |  |
| HTN | no | N(%) | 1(3.6) | 27(96.4) | 0.048 |
|  | yes | N(%) | 3(30.0) | 7(70.0) |  |
| comorbidities | no | N(%) | 1(5.0) | 19(95.0) | 0.328 |
|  | yes | N(%) | 3(16.7) | 15(83.3) |  |
| smoking | no | N(%) | 2(14.3) | 12(85.7) | .088 |
|  | ex | N(%) | 2(28.6) | 5(71.4) |  |
|  | current | N(%) | 0(0.0) | 16(100.0) |  |
| symptoms.1.cough | no | N(%) | 1(9.1) | 10(90.9) | 1 |
|  | yes | N(%) | 3(11.1) | 24(88.9) |  |
| sym.chest.pain | no | N(%) | 3(11.1) | 24(88.9) | 1 |
|  | yes | N(%) | 1(9.1) | 10(90.9) |  |
| symp.haemoptysis | no | N(%) | 3(10.0) | 27(90.0) | 1 |
|  | yes | N(%) | 1(12.5) | 7(87.5) |  |
| Symp.shortness.of.breath | no | N(%) | 2(12.5) | 14(87.5) | 1 |
|  | yes | N(%) | 2(9.1) | 20(90.9) |  |
| sympt.pathologi.fracture | no | N(%) | 2(6.5) | 29(93.5) | 0.147 |
|  | yes | N(%) | 2(28.6) | 5(71.4) |  |
| pathological.type | adenocarcinoma | N(%) | 2(12.5) | 14(87.5) | .594 |
|  | large cell neuroendocrine carcinoma | N(%) | 0(0.0) | 1(100.0) |  |
|  | squamous cell carcinoma | N(%) | 2(18.2) | 9(81.8) |  |
|  | SCLC | N(%) | 0(0.0) | 10(100.0) |  |
| side | right | N(%) | 1(3.8) | 25(96.2) | 0.057 |
|  | left | N(%) | 3(30.0) | 7(70.0) |  |
| site | hilar | N(%) | 1(6.7) | 14(93.3) | .698 |
|  | upper and lower lobes | N(%) | 0(0.0) | 3(100.0) |  |
|  | upper | N(%) | 2(18.2) | 9(81.8) |  |
|  | lower | N(%) | 1(16.7) | 5(83.3) |  |
|  | chest wall mass | N(%) | 0(0.0) | 1(100.0) |  |
| ct.pleural.effusion | no | N(%) | 3(14.3) | 18(85.7) | 0.613 |
|  | yes | N(%) | 1(5.9) | 16(94.1) |  |
| ct.lymphnodes | no | N(%) | 1(11.1) | 8(88.9) | 1 |
|  | yes | N(%) | 3(10.3) | 26(89.7) |  |
| pulmonary.metastatic.nodules | no | N(%) | 1(4.8) | 20(95.2) | 0.307 |
|  | yes | N(%) | 3(17.6) | 14(82.4) |  |
| collapse | no | N(%) | 4(13.8) | 25(86.2) | 0.554 |
|  | yes | N(%) | 0(0.0) | 9(100.0) |  |
| ct.chestwall.mass | no | N(%) | 4( ) | 29(87.9) | 1 |
|  | yes | N(%) | 0(0.0) | 5(100.0) |  |
| bone.metas | no | N(%) | 0(0.0) | 22(100.0) | 0.025 |
|  | yes | N(%) | 4(25.0) | 12(75.0) |  |
| brain.mets | no | N(%) | 4(12.1) | 29(87.9) | 1 |
|  | yes | N(%) | 0(0.0) | 5(100.0) |  |
| peripheral.ln | no | N(%) | 4(12.5) | 28(87.5) | 1 |
|  | yes | N(%) | 0(0.0) | 6 (100.0 ) |  |
| liver.metastasis | no | N(%) | 3(11.1) | 24(88.9) | 1 |
|  | yes | N(%) | 1(9.1) | 10(90.9) |  |
| suprarenal.mets | no | N(%) | 1(3.2) | 30(96.8) | 0.010 |
|  | yes | N(%) | 3(50.0) | 3(50.0) |  |
| surgery | no | N(%) | 4(11.1) | 32(88.9) | 1 |
|  | yes | N(%) | 0(0.0) | 1(100.0) |  |
| course.group | stationary/regressive | N(%) | 0(0.0) | 21(100.0) | 0.048 |
|  | progressive | N(%) | 3(23.1) | 10(76.9) |  |
| t.gp | T1/2 | N(%) | 2 (18.2 ) | 9(81.8) | 0.564 |
|  | T3/4 | N(%) | 2(7.4) | 25(92.6) |  |
| n.gp | 0/1 | N(%) | 1 (10.0 ) | 9(90.0) | 1 |
|  | 2/3 | N(%) | 3(10.7) | 25(89.3) |  |
| M.GP | m0 | N(%) | 0(0.0) | 6(100.0) | 1 |
|  | M1A,B,C | N(%) | 4(12.5) | 28(87.5) |  |
| stage.gp | II,III | N(%) | 0(0.0) | 5(100.0) | 1 |
|  | IV | N(%) | 4(12.1) | 29(87.9) |  |
